# Morvan Syndrome Secondary to Thymic Carcinoma in a Patient with Systemic Lupus Erythematosus

**DOI:** 10.1155/2016/9142486

**Published:** 2016-05-10

**Authors:** Gabrielle Macaron, Elie El Rassy, Salam Koussa

**Affiliations:** Department of Neurology, Hotel-Dieu de France University Hospital, Faculty of Medicine, Saint Joseph University, P.O. Box 166830, Beirut, Lebanon

## Abstract

Morvan syndrome (MoS) is a rare paraneoplastic autoimmune disorder characterized by peripheral nerve hyperexcitability, autonomic dysfunction, and sleep disorders. Systemic lupus erythmatosus (SLE) cooccurs in 6–10% of patients with thymoma. It may occur before, concurrently with, or after thymoma diagnosis. This paper reports the first case of cooccurrence of SLE, thymic carcinoma, and MoS. The cooccurrence of SLE, thymoma, and MoS delineates the generalized autoimmunity process. Symptoms of both MoS and SLE abated upon tumor resection.

## 1. Introduction

Morvan syndrome (MoS) is a rare autoimmune condition that was first described in 1890 with data limited to case reports and small case series [1]. It has male predilection with usually subacute to chronic presentation often secondary to tumors [2, 3]. MoS is often a paraneoplastic condition associated with thymoma. Systemic lupus erythematosus (SLE) can precede or occur concurrently with or after thymoma diagnosis with a prevalence of 6–10% with very few cases considered paraneoplastic [4, 5]. This paper reports the first case of cooccurrence of SLE, thymic carcinoma, and MoS.

## 2. Case Report

We report the case of an otherwise healthy 52-year-old patient except for SLE diagnosed six years earlier upon the presence of arthritis of the proximal interphalangeal joints, photosensitive rash of the face and extremities, anemia, leucopenia, and high titers of ANA and anti-ds DNA antibodies. The patient was successfully treated with Prednisone 1 mg/kg/d and Hydroxychloroquine 200 mg PO BID but voluntarily discontinued her treatment in 2009 and was lost to follow-up.

In early 2015, the patient presented to our department with recurrent generalized convulsions refractory to Valproic Acid 500 mg PO BID. She reported the onset of progressive irritability that started two weeks prior to her consultation. Other symptoms included disordered sleep, intermittent nocturnal delusions, insomnia, and dream enactment. She also noted new onset of generalized pruritus, hypersialorrhea, hyperhidrosis of palms, and distal paresthesias in upper and lower limbs. Sleep disturbances were prominent, with marked nocturnal confusion and delusions. Her clinical examination was only relevant for intermitted episodes of agitation and confusion. Her labs showed a plasma sodium level of 113 mEq/L, plasma osmolarity of 246 mEq/L, urine osmolarity of 246 mOsm/L, and urine sodium level of 296 mOsm/L. Other laboratory tests including hemoglobin, platelets, creatinine, liver enzymes, TSH, vitamin B12, PPD, TPHA and VDRL, ANA blot, and anti-cardiolipine, anti-transaminase, anti-endomysial, and anti-gliadin antibodies were within normal values. ANA titer was 1/1280, Anti-ds DNA 261.4 IU/mL (normal values < 100 IU/mL), C3 75.3 mg/100 mL (normal values: 90–180 mg/100 mL), and C4 7.2 mg/100 mL (normal values: 10–40 mg/100 mL) (Table 1). Cerebrospinal fluid (CSF) proteins, cells, and IgG were within normal limits. Chest X-ray, brain MRI, and brain MRA were noncontributory. Electroencephalogram (EEG) and electroneuromyogram (ENMG) were normal. Subsequently, the hyponatremia was adjusted and the Valproic Acid was switched to Levetiracetam with progressive tapering.

**Figure 1:**
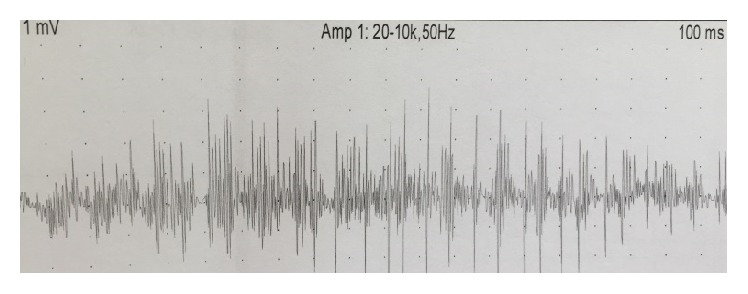
EMG showing spontaneous discharges in the left rectus femoris.

**Figure 2:**
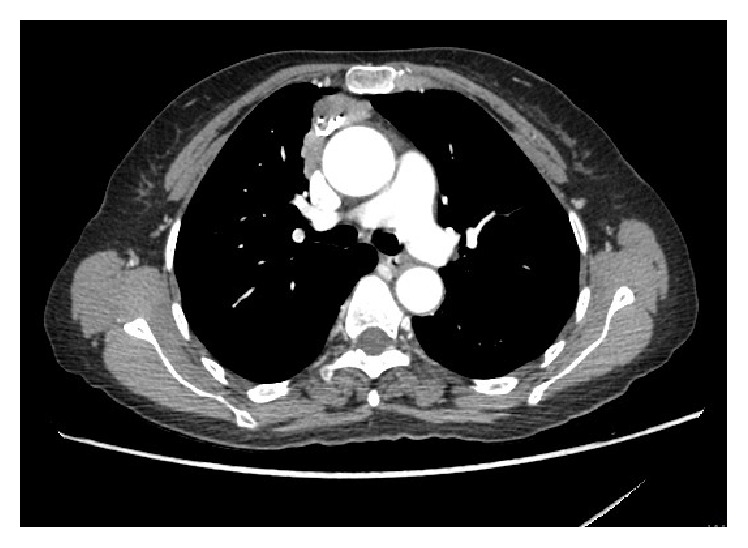
Chest CT showing anterior mediastinal mass with calcification and regional invasion consistent with athymic tumor.

One week later, she presented two more generalized convulsions with new onset of continuous, irregular, undulating muscle movements in the proximal lower extremities and the chin. Brain imaging was unchanged. Sodium level was normal (140 mEq/L), and C3 and C4 levels were lower than a week earlier (59.7 and 7.1 mg/100 mL, resp.) (Table 1). CSF control was normal. ENMG showed myokymic discharges, fibrillation potentials, and positive sharp waves in rectus femori, tibialis anterior, biceps brachii, and mentalis muscles (Figure 1). EEG showed diffuse moderate slowing of background activity, without epileptic discharges. A contrast-enhanced chest CT scan showed a calcified anterior mediastinal mass with pleural, pericardial, and diaphragmatic invasion (Figure 2). A CT guided biopsy of the mass confirmed the diagnosis of type B3 thymoma (well-differentiated thymic carcinoma, Masaoka stage IVa). Anti-caspr2 antibodies were positive in the plasma and negative in the CSF, whereas anti-Lgi1 antibodies were negative in the serum and CSF (Table 1). The diagnosis of paraneoplastic MoS was retained. Surgical resection of the thymic mass was performed followed by reintroduction of Prednisone 0.5 mg/kg/d and Hydroxychloroquine 200 mg PO BID. Neuropsychiatric symptoms, myokymia, itching, and hypersialorrhea completely resolved after 48 hours. Three weeks after surgery, ANA level was 1/160, Anti ds DNA was 150 U/mL, and C3 and C4 level were within normal limits (Table 1). Two months later, all symptoms resolved and neurological examination was normal.

## 3. Discussion

Although MoS was never described in patients with SLE, its pathophysiology is largely determined by the generalized autoimmunity seen in patients with an autoimmune disease [6, 7]. On the other hand, MoS may be associated with several tumors, of which thymoma is the most common. Interestingly, when a thymoma is present, myasthenia gravis and acetylcholine receptor antibodies frequently coexist with MoS [2, 3].

Pathophysiologically, MoS is associated with voltage-gated potassium channel- (VGKC-) complex antibodies and it is linked to a spectrum of diseases comprising MoS, neuromyotonia (NMT), limbic encephalitis (LE), and acquired epilepsy, for instance, faciobrachial dystonic seizures [2]. VGKC-complex antibodies are directed toward leucine-rich glioma inactivated 1 (Lgi1) localized in hippocampal structures and contactin-associated protein like 2 (caspr2) identified in the juxtaparanodal region of myelinated axons of the peripheral nervous system as well as the hippocampus. Lgi1 antibodies, mainly found in patients with LE or idiopathic epilepsy, are generally not associated with tumors. Lgi1 antibodies are also associated with hyponatremia secondary to SIADH due to their binding to hypothalamic paraventricular nucleus neurons. Caspr2 antibodies, found in MoS and NMT, are often associated with thymoma and less often with paraneoplastic LE [2, 8, 9].

Although our patient technically fulfills the criteria for MoS diagnosis, her central nervous system symptoms were initially attributed to SLE and electrolyte disturbance whereas her mild nerve hyperexcitability and autonomic findings were due to Caspr2 antibody. Thus, the diagnosis of MoS in our patient was misled by the initial presentation and mildness of her symptoms. Effectively, large cohorts of MoS patients report severe symptoms of neuromyotonia, painful cramps, and major hyperexcitability findings. We only retained the MoS diagnosis upon the later constellation of symptoms including appearance of peripheral hyperexcitability, dysautonomia, and sleep disturbances. Although not pathognomonic, insomnia and generalized tonicoclonic seizures are seen, respectively, in 90% and 35% of cases [2, 3]. On the other hand, the later symptomatic occurrences of peripheral hyperexcitability and autonomic dysfunction are more suggestive of MoS. Peripheral nerve involvement is usually the presenting presentation with symptoms of hyperexcitability, neuropathic pain, and sensory loss in the lower extremities and areflexia [2, 3]. Autonomic dysfunction, reported in 93.1% of patients, includes hyperhidrosis, the most frequent finding, followed by cardiovascular instability, constipation, hypersialorrhea, and urinary disorders [2, 3]. Other features include amnesia, hallucinations, delusions, agitation, confusion, generalized pruritus, fever, anorexia, hyperphagia, and laryngeal myotonia [2, 3]. One limitation of this report is the absence of a sleep study; however, our patient presented with nocturnal delusions and insomnia and satisfied the clinical criteria for REM-sleep behavior disorder, as she had repetitive, sometimes injurious episodes of complex motor behavior and speech during sleep without confusion on awakening.

The clinical and paraclinical amelioration of SLE in our patient following thymectomy is noteworthy. However, the effect of thymectomy on SLE is unclear. The vast majority of papers report an occurrence or exacerbation of SLE after thymectomy except for one case [4]. In fact, the effect of thymectomy on the induction and/or modification of SLE was studied in mice with results indicating that thymectomy prevented disease only in a particular mice strain but it did not induce SLE in normal mice [10]. Unfortunately, the clinical significance of this finding is yet to be determined. Such patients warrant prospective follow-up to identify factors that would predict the effect of thymectomy on SLE.

The cooccurrence of SLE, thymoma, and MoS supports the polyautoimmunity of our patient and the diagnosis of multiple autoimmune syndromes [11]. The rapid resolution of the neurological symptoms after thymoma resection is in favor of the paraneoplastic status of MoS and possibly SLE. However, paraneoplastic SLE is of very rare occurrence and to our knowledge our case would be the second case report in literature; thus such assumption would be hypothetical and clarified by a longer follow-up.

## Figures and Tables

**Table 1:** Laboratory results.

	Before thymectomy	Three weeks after thymectomy
Anti-caspr2^*∗*^	Positive	Not tested
anti-Lgi1^*∗*^	Negative	Not tested
ANA	1/1281	1/160
Anti ds DNA (IU/mL)	261.4	150
C3 (mg/100 mL)^*∗∗*^	75.3	89
	59.7	
C4 (mg/100 mL)^*∗∗*^	7.2	20
	7.1	

^*∗*^Serum anti-caspr2 antibodies were positive; CSF anti-caspr2, serum, and CSF anti-Lgi1 antibodies were negative.

^*∗∗*^Two values before thymectomy tested at 1-week interval.

## References

[1] Waluskinski O., Honnorat J. (2012). Augustin Morvan (1819–1897) a littleknown rural physician and neurologist. *Revue Neurologique*.

[2] Irani S. R., Pettingill P., Kleopa K. A. (2012). Morvan syndrome: clinical and serological observations in 29 cases. *Annals of Neurology*.

[3] Abou-Zeid E., Boursoulian L. J., Metzer W. S., Gundogdu B. (2012). Morvan syndrome: a case report and review of the literature. *Journal of Clinical Neuromuscular Disease*.

[4] Boonen A., Rennenberg R., van der Linden S. (2000). Thymoma-associated systemic lupus erythematosus, exacerbating after thymectomy. A case report and review of the literature. *Rheumatology*.

[5] Shelly S., Agmon-Levin N., Altman A., Shoenfeld Y. (2011). Thymoma and autoimmunity. *Cellular & Molecular Immunology*.

[6] Shih W. H., Landau M. E., Barner K. C., Campbell W. W. (2003). Acquired neuromyotonia in association with systemic lupus erythematosus. *Journal of Clinical Neuromuscular Disease*.

[7] Taylor P. W. (2005). Isaacs' syndrome (autoimmune neuromyotonia) in a patient with systemic lupus erythematosus. *The Journal of Rheumatology*.

[8] Lancaster E., Huijbers M. G. M., Bar V. (2011). Investigations of caspr2, an autoantigen of encephalitis and neuromyotonia. *Annals of Neurology*.

[9] Irani S. R., Alexander S., Waters P. (2010). Antibodies to Kv1 potassium channel-complex proteins leucine-rich, glioma inactivated 1 protein and contactin-associated protein-2 in limbic encephalitis, Morvan's syndrome and acquired neuromyotonia. *Brain*.

[10] Hang L., Theofilopoulos A. N., Balderas R. S., Francis S. J., Dixon F. J. (1984). The effect of thyemctomy on lupus-prone mice. *The Journal of Immunology*.

[11] Cojocaru M., Cojocaru I. M., Silosi I. (2010). Multiple autoimmune syndrome. *Maedica*.

